# Personal Growth and Well-Being in the Time of COVID: An Exploratory Mixed-Methods Analysis

**DOI:** 10.3389/fpsyg.2021.648060

**Published:** 2021-03-24

**Authors:** Juensung J. Kim, Melanie Munroe, Zhe Feng, Stephanie Morris, Mohamed Al-Refae, Rebecca Antonacci, Michel Ferrari

**Affiliations:** Department of Applied Psychology and Human Development, Ontario Institute for Studies in Education, University of Toronto, Toronto, ON, Canada

**Keywords:** coronavirus, physical distancing, self-transcendence, well-being, coping

## Abstract

The physical distancing measures necessitated by COVID-19 have resulted in a severe withdrawal from the patterns of daily life, necessitating significantly reduced contact with other people. To many, such withdrawal can be a major cause of distress. But, to some, this sort of withdrawal is an integral part of growth, a pathway to a more enriching life. The present study uses a sequential explanatory QUAN-qual design to investigate whether people who felt that their lives had changed for the better after being forced to engage in physical distancing, what factors predicted such well-being, and how they spent their time to generate this sense of well-being. We invited 614 participants who reported closely following physical distancing recommendations to complete a survey exploring this topic. Our analyses, after controlling for all other variables in the regression model, found a greater positive association between presence of meaning in life, coping style, and self-transcendent wisdom and residualized current well-being accounting for retrospective assessments of well-being prior to physical distancing. An extreme-case content analysis of participants' personal projects found that participants with low self-transcendent wisdom reported more survival-oriented projects (e.g., acquiring groceries or engaging in distracting entertainments), while participants reporting high self-transcendent wisdom reported more projects involving deepening interactions with other people, especially family. Our findings suggest a more nuanced pathway from adversity to a deeper sense of well-being by showing the importance of not merely coping with adversity, but truly transcending it.

## Personal Growth and Well-Being in the Time of COVID: An Exploratory Mixed-Methods Analysis

The physical distancing measures necessitated by the COVID-19 pandemic have resulted in a massive withdrawal from the habitual patterns of daily life and in significantly less contact with other people. To many, such withdrawal can lead to disconnection and loneliness, a major cause of stress, anxiety, depressive symptoms, self-harm, and suicidal ideation (Goldsmith et al., 2002; Cacioppo et al., 2006; Meltzer et al., 2013). Research on the effects of current lockdown and physical distancing protocols has already begun to ring alarm bells. Physical distancing policies have been found to have overall negative effects on the mental health of young adults in China, exacerbated by a steady stream of dire news about the pandemic (Chen et al., 2020). Participant samples from California and the UK have reported feeling more hopeless and less engaged than a comparison group of first-time incarcerated inmates (Dhami et al., 2020). Furthermore, increased rates of substance use have also been seen as result of loneliness and isolation as individuals are using substances to cope with the emotional impact of the pandemic (Czeisler et al., 2020). Loneliness affects people of all ages, genders, and socioeconomic status. A study that examined loneliness as a predictor of mental health outcomes found that individuals experienced an increase in mental health symptom severity and decreased recovery and quality of life scores (Wang et al., 2020). Such effects appear to be heightened among already vulnerable population, with loneliness being found to be particularly detrimental to the mental health of certain vulnerable societal groups such as seniors, migrants, asylum seekers and those in the LGBT population (Eres et al., 2020); often the same populations most at risk as a result of the current pandemic.

Prior to the pandemic, North America was already beginning to demonstrate a worrying culture of disconnection, with people feeling increasingly isolated. The meteoric rise in single-person households over the past decade was accompanied by an increase in feelings of loneliness and disconnection (Snell, 2017), along with a marked increase in major depressive episodes among those born in both the 1990's and 1950's (Twenge et al., 2019). Religious institutions, formerly a major source of interpersonal connection and meaning-making (Park et al., 2013), have seen a steady decline in membership, which Eberstadt (2013) directly ties to more people living alone. While loneliness and disconnection were already a topic of concern, the pandemic, fueling further separation of family units and closure of churches in many parts of the world, is likely to have exacerbated an already worrying trend. Or has it?

While loneliness is well-known to have negative impacts on many aspects of mental and physical health, solitude, or at least some form of withdrawal from mundane activities, is a necessary part of achieving higher forms of development in many religious and spiritual traditions, from the Desert Fathers of Christianity to the Sannyasis of Hinduism. The Buddhist Dhammapadda, likewise, uses the image of the elephant walking alone through the jungle to symbolize the often-solitary path of the aspirant in search of enlightenment. Modern qualitative research with spiritual aspirants (Thomas, 1991; Durà-Vilà and Leavey, 2017) finds that they consider solitude an integral part of their pursuit of wisdom, a way for them to engage in uninterrupted reflection and meditation. Barbour (2004, 2013) has argued that aloneness is an intrinsically spiritual value; seeking separation from the ordinary rhythms of societal life is a time-honored way to open oneself up to greater sources of connection and meaning. Averill and Sundararajan (2014) argue that the spiritual value of solitude comes from a relational soft reset, allowing one to focus on cultivating one's relationships with higher or greater sources of meaning. Solitude is often pursued as a strategy for affective self-regulation, a way to recover from intense emotional experiences (Nguyen et al., 2018).

The benefits of solitude for personal and spiritual growth, however, make two key assumptions: that solitude is both voluntary and used constructively. This is particularly clear in studies of aloneness in children: while self-determined solitude is consistently associated with positive developmental outcomes, non-self-determined solitude is associated with greater loneliness and its accompanying long-term problems (Galanaki, 2013; van Zyl et al., 2018; Corsano et al., 2020). Regarding the use of solitude, Lay et al. (2019) found that trait self-reflection, by definition involuntary, was more strongly associated with negative solitude experiences than positive, suggesting a greater opportunity for moments of despair when forced to be alone with one's thoughts. However, Weststrate (2019) found that deliberate exploratory reflection on one's life is a vital component in developing wisdom and a meaningful life.

As humans are inherently relational creatures, involuntary solitude may open up greater opportunities for individuals to experience an existential vacuum [see Frankl (1963), Garfield (1973), Reker et al. (1987)]. Weinstein et al. (2012) observe that the notion of existential vacuum in existential psychology, as well as the phenomenon of pervasive motivation in literature on self-determination theory, are associated with a sort of epistemological loneliness, a feeling of being mentally alone. Without strong relationships in place, or the capacity to build them, the sense of aloneness can become unbearable. Those who do have such strong relationships with others, or with greater things, likely have less cause for concern. People with stronger relationships tend to weather involuntary solitude better (Pauly et al., 2018), as do those who have built a healthy relationship with solitude itself, by being more likely to seek it out (Lay et al., 2019). Lay et al. (2019) also found that greater social self-efficacy was another good predictor of positive experiences of solitude, which helps reconcile Wei (2020) finding that introverts are suffering more than expected under lockdown conditions in the United States. It takes a good deal of self-confidence to be alone; under present conditions, an aptitude for it may not be enough.

As a result of COVID-19, humanity is currently experiencing involuntary solitude on an unprecedented scale. However, as per the tenets of existential positive psychology (Wong, 2020), times of such great suffering also provide the greatest opportunities for growth. Looking to history, we have some reason to be optimistic—or perhaps tragically optimistic (Wong and McDonald, 2002; Leung, 2019). Loneliness became an important factor during the 2003 SARS epidemic, as well, but resulted in increased social cohesion due to the detrimental impact of isolation (Lau et al., 2005; Saltzman et al., 2020). Even in the early stages of the pandemic, during the spring and summer of 2020, a large-scale narrative inquiry conducted in Italy found that some participants were already beginning to represent the pandemic as a time for re-evaluation of personal and social priorities, not just as a crisis (Venuleo et al., 2020).

According to the MORE Wisdom Model (Weststrate and Glück, 2017; Weststrate, 2019; Glück, in press), “critical life experiences”—typically disruptive to life as usual—are important to fostering the development of wisdom, when accompanied by the exploratory reflective processing. This is very much in line with prominent models of post-traumatic growth, most notably the model championed by Tedeschi and Calhoun (1996, 2004; Blevins and Tedeschi, in press), according to which post-traumatic growth is a process of self-transformation involving the reconstruction of an individual's basic assumptions about how life ought to be lived to promote future flourishing. Paradoxically, post-traumatic growth involves: a greater appreciation for one's life, a newfound sense of personal strength, improved relationships, and consequently, greater wisdom. Indeed, with the right perspective, adverse events causing suffering can be transformed in the moment, potentially ameliorating the negative effect during, rather than after, the ongoing traumatic event (Leung, 2019; Mead et al., 2020; Wong, 2020).

Thus, the isolation caused by the pandemic gives us cause for both concern and optimism. On the one hand, such isolation can and does impact most people negatively. On the other hand, strong relationships can help people weather such times of suffering, and such disruptions offer the opportunity to take stock of one's world and re-evaluate one's priorities. As per PP2.0 (Wong, 2020), if one is able to take this time of suffering and necessary solitude, affirm it, and use it to grow, one can come to experience greater meaning and well-being than before such trials—an understanding that has a long history in philosophy and clinical psychology (Halling and Nill, 1995). If anyone is lucky enough to feel that they have grown as a result of the current pressures to self-isolate, it is likely people who have focused on the key things in life: forging connections and finding meaning. If this is how some people have been spending this time, they have spent this time wisely.

The present exploratory mixed-methods study began with these two questions: (1) How do North Americans feel they have been doing since the introduction of physical distancing measures, and (2) how are they spending their time? Using an explanatory QUAN-qual design, this paper describes and elucidates our finding that it is the relationships we build, and the things we care about, that lead to a life of flourishing, even in the face of global suffering.

## Method

This study was approved by the Research Ethics Board at the University of Toronto. Workers on Amazon's Mechanical Turk chose to participate in our human intelligence task which was advertised as a study on coping with the COVID-19 pandemic. Workers were directed to a link to participate in our online survey, hosted on Qualtrics. All participants gave informed consent before starting the survey. In part one, a screening was made in which participants indicated the extent of their engagement in physical distancing practices before completing a series of measurements in which they were instructed to respond while reflecting on their life prior to physical distancing. These included instruments on well-being, personal wisdom, presence and search for meaning, coping styles, negative emotion, and alienation.

Participants who reported closely following physical distancing recommendations were invited to complete a second stage of the survey, in which participants completed many of the same measures for a second time, this time reflecting on their life and experiences as they were at the time of completion (see Table 1 for list of measures completed in each section of the survey). They also completed additional measures of self-transcendent wisdom and engagement in personal projects. This two-stage design and broad array of measurements were intended to capture participants' experiences of physical distancing, their motivations for doing so, how they felt life has changed under distanced conditions, and why. Participants were not aware that their responses from the pre-physical distancing measures would be compared with their responses on the later set of measures related to the current time of completion.

**Table 1:** List of measures completed in each section of the survey.

**Measurements**	**Part 1**	**Part 2**
Multidimensional Scale of Perceived Social Support (MSPSS)	X	
12-item Abbreviated Three-Dimensional Wisdom Scale (Brief3DWS)	X	
Meaning in Life Questionnaire (MLQ)	X	X
PERMA-profiler	X	X
Personal Projects Analysis Workbook	X	X
Adult Self-Transcendence Inventory (ASTI)		X
Brief COPE Inventory		X

### Participants

Data was collected from mid-April to end-of-July, 2020, through a two-part online survey distributed *via* Amazon's Mechanical Turk platform to residents of Canada and the U.S.A. To ensure the quality of our data, we implemented many approaches in the collection of our responses from Amazon's Mechanical Turk platform and screened participant data for problematic responses. On Mechanical Turk, workers were required to have a “human intelligence task” (HIT) approval rating ≥90, later upgraded to ≥95, along with having ≥100 HITs previously approved. We also included two attention checks (e.g., “select _ for this item”), one in each half of the survey. Workers who failed all attention checks that they came across were excluded from the study (n.b., individuals who did not pass the pre-established physical distancing cut-off score only encountered one attention check). IP duplications in submitted responses were screened and examined^1^. Open-ended questions within the Personal Projects Analysis Workbook (measure detailed in the following section) were assessed by two graduate coders for problematic responses, and cases that contain copy-paste responses or answers that did not relate to the prompt were removed from the study. Lastly, participants who scored the same responses for all items across several measurements (i.e., having standard deviations equal to 0 across several measurements) were removed before data analysis.

The final sample of physically-distanced participants consists of 274 Americans and 340 Canadians (*N* = 614), with 279 females and 334 males with a mean age of 35 (see Table 2). Of these participants, 50% had a bachelor's degree or equivalent. Most household income were between C$40,000 and C$60,000. The mean number of children participants had was 0.78 (Table 2).

**Table 2:** Demographics (*N* = 614).

**Variables**	***n***	**%**
**Nation**		
Canada	339	55.21
United States of America	275	44.79
**Gender**^***a***^		
Male	334	54.40
Female	279	45.44
**Age**		
18–64	592	96.42
65 and over	22	3.58
**Religious affiliation**^***a***^		
Christian	248	40.39
Buddhist	14	2.28
Muslim	29	4.72
Hindu	18	2.93
Atheist	124	20.20
Agnostic	137	22.31
Other	42	6.84
**Marital status**		
Never married	264	43.00
Married/living with a partner	304	49.51
Divorced/separated	40	6.51
Widowed	6	0.98
**Number of children**		
None	374	60.91
One child	89	14.50
Two children	94	15.31
More than two children	57	9.28
**Highest level of education**		
High school diploma and below	181	29.48
Bachelor's degree or equivalent	322	52.44
Graduate degree or equivalent	111	18.08
**Household income for 2019**		
Under C$20,000	47	7.65
Between C$20,000 and C$40,000	110	17.92
Between C$40,000 and C$60,000	118	19.22
Between C$60,000 and C$80,000	99	16.12
Between C$80,000 and C$100,000	90	14.66
Between C$100,000 and C$150,000	91	14.82
Over C$150,000	59	9.61

a *n = 612 with two missing values*.

### Measures

#### Physical Distancing

Participants were asked how much they were participating in physical distancing measures by staying home, avoiding visiting others or having others visit them at home, avoiding religious gatherings and other social events, limiting trips to the grocery stores, or outdoor activities, and maintaining a safe distance from people who have traveled, in stores, or showing COVID-related symptoms; also whether they were canceling travel plans, quarantining after travel, self-isolating due to symptoms or close contact with people who had then, and generally following public health guidance about COVID as much as possible. Participants rated the extent to which they did so on a scale from 0 (Not at all) to 10 (To my best ability). Participants who scored an average of eight or above on all items were permitted to fill out additional questionnaires, as we were only interested in those who were physically distancing most of the time. Only participants who scored an average of eight or above on our measure of physical distancing are included in the present study.

#### Religiosity and Spirituality

Participants were asked to rate on a scale from 0 (Not at all religious/spiritual) to 10 (Very religious/spiritual) how religious or spiritual they were, in order to measure both the degree of religiosity and the degree of spirituality separately.

#### Coping

The Brief COPE Inventory (Carver, 1997) was used to measure coping styles used after physical distancing was enacted. This measure consists of 28 items with 14 subscales: (1) self-distraction (e.g., “I've been turning to work or other activities to take my mind off things”), (2) active coping (e.g., “I've been concentrating my efforts on doing something about the situation I'm in”), (3) denial (e.g., “I've been saying to myself "this isn't real”), (4) substance use (e.g., “I've been using alcohol or other drugs to make myself feel better”), (5) emotional support (e.g., “I've been getting emotional support from others”), (6) instrumental support (e.g., “I've been getting help and advice from other people”), (7) behavioral disengagement (e.g., “I've been giving up trying to deal with it”), (8) venting (e.g., “I've been saying things to let my unpleasant feelings escape”), (9) positive reframing (e.g., “I've been trying to see it in a different light, to make it seem more positive”), (10) planning (e.g., “I've been trying to come up with a strategy about what to do”), (11) humor (e.g., “I've been making jokes about it”), (12) acceptance (e.g., “I've been accepting the reality of the fact that it has happened”), (13) religion (e.g., “I've been trying to find comfort in my religion or spiritual beliefs”), and (14) self-blame (e.g., “I've been criticizing myself”). Each item is rated on a 4-point Likert scale, ranging from 1 (I haven't been doing this at all) to 4 (I've been doing this a lot). In our study, overall Cronbrach's alpha was good (Alpha = 0.84).

#### Personal Wisdom

The 12-item Abbreviated Three-Dimensional Wisdom Scale (3D-WS-12; Thomas et al., 2017) was used to measure personal wisdom. This scale selects items from Ardelt's (2003) original scale, which includes three dimensions: cognitive (e.g., “I try to anticipate and avoid situations where there is a likely chance I will have to think in depth about something”), compassionate (“Sometimes I feel a real compassion for everyone”), and reflective (e.g., “I either get very angry or depressed if things go wrong”). Scores were rated on a 5-point Likert scale, ranging from 1 (Definitely true of myself) to 5 (Not true of myself). Total scores are calculated by taking the mean of all items on the scale. Cronbach's alpha for the 3D-WS-12 was good (Alpha = 0.81).

#### Social Support

To measure participants' perceptions of social support, we used the Multidimensional Scale of Perceived Social Support (MSPSS; Zimet et al., 1988). This measure consists of 12 items and three subscales: (1) Significant other (e.g., There is a special person who is around when I am in need), (2) Family, (e.g., I can talk about my problems with my family), and (3) Friends (e.g., My friends really try to help me). All items were answered on a 7-point Likert scale ranging from 1 (Very strongly disagree) to 7 (Very strongly agree). Total scores are calculated by taking the mean across the raw score from each item. Cronbach's alpha for the MSPSS was excellent (Alpha = 0.94).

#### Well-Being

The PERMA-profiler (Butler and Kern, 2016), was administered to measure participants' well-being. The PERMA-profiler assesses well-bring across five subscales, also known as the five pillars of well-being: positive emotion, engagement, relationships, meaning, and accomplishment. The final well-being score is the mean across all PERMA subscale items. To determine if participants perceived their well-being as being different during and prior to physical distancing, participants completed both retrospective and current versions of the measure. Total scores and subscale scores were calculated for both pre-pandemic and current moment. Cronbach's alpha for the well-being subscale of the PERMA pre-pandemic and at the current moment were excellent at 0.96. The PERMA also includes as negative emotion subscale, whose Cronbach's alphas were good at 0.83 (pre-pandemic) and 0.85 (at the current moment). The loneliness subscale contains only one item, so Cronbach's alpha cannot be computed.

#### Meaning in Life

The Meaning in Life Questionnaire (MLQ; Steger et al., 2006) was used to measure both search for, and presence of, meaning in life before physical distancing (pre-pandemic) and once physical distancing had been enacted (at the current moment during the pandemic). The measure consists of 10 items, such as “I am looking for something that makes my life feel meaningful” (search for meaning, 5 items) and “I understand my life's meaning” (presence of meaning, 5 items). Participants rated these items on a 7-point Likert scale, ranging from 1 (absolutely untrue) to 7 (absolutely true). To determine if participants perceived their experienced sense of meaning in life as being different during and prior to physical distancing, participants completed both retrospective and current versions of the measure Total scores for search and presence of meaning were calculated separately and scores for pre-pandemic, current moment, and a pre-post difference score were calculated for each subscale. Cronbach's alpha for the search for meaning at 0.94 (pre-pandemic) and 0.95 (at the current moment), and presence of meaning at 0.94 (pre-pandemic) and 0.90 (at the current moment), were all excellent.

#### Self-Transcendent Wisdom

The Adult Self-Transcendence Inventory (ASTI; Levenson et al., 2005) was used to measure wisdom as self-transcendence since the beginning of physical distancing (self-reported at the present moment). The ASTI broadly defines self-transcendence as decreased egoic self-saliency and increased sense of connectedness. It consists of 29 items with six subscales (Koller et al., 2017). Five subscales measure self-transcendent wisdom: (1) Self-Knowledge and Integration (e.g., “Since physical distancing was enacted, I have better sense of humor about myself”); (2) Peace of Mind (e.g., “Since physical distancing was enacted, I am more often engaged in quiet contemplation”); (3) Non-Attachment (e.g., “Since physical distancing was enacted, I have become less concerned about other people's opinions of me”); (4) Self-Transcendence (e.g., “Since physical distancing was enacted, I feel that my individual life is a part of a greater whole”); and (5) Presence in the Here-and-Now and Growth (e.g., “Since physical distancing was enacted, I find more joy in life”). Participants rated on a 4-point Likert scale, ranging from 1 (Disagree strongly) to 4 (Agree strongly). Total scores were calculated by taking the mean of all individual items in the self-knowledge and self-integration, peace of mind, non-attachment, self-transcendence, and presence in the here-and-now and growth subscales, as recommended for measuring overall self-transcendent wisdom. The 6th scale, Alienation (e.g., “Since physical distancing was enacted, I feel that my life has less meaning”), was treated as an individual subscale in all analyses, following Koller et al. (2017). Cronbach's alpha for the total score was excellent (Alpha = 0.94), and for alienation was acceptable (Alpha = 0.65). Unlike our other measures, the ASTI is designed to assess the difference between the present moment and some previously specified time. As such, participants only filled out a single version of the ASTI.

#### Personal Projects

Finally, the Personal Projects Analysis Workbook (PPAW; Little, 1983) Module 1 was used to solicit participants' personal projects; that is, their most important goals and activities. Personal projects analysis conceptualizes the individual as collections of ongoing personal projects, as opposed to collections of personality traits. The PPAW Module 1 invites participants to list as many ongoing projects as comes to their mind—from the immediately utilitarian (e.g., “Mow the lawn”) to the most abstract (e.g., “Clarify my philosophy of life”). In separate sections of the survey, participants were asked to generate separate lists of personal projects both prior to and during physical distancing.

### Analytic Strategy

#### Latent Profile Analysis

To reduce the number of variables in later regression analyses, we conducted a latent profile analysis (LPA) on the 14 coping subscales from the Brief COPE (self-distraction, active coping, denial, substance use, emotional support, instrumental support, behavioral disengagement, venting, positive reframing, planning, humor, acceptance, religion, and self-blame) to identify latent subgroups of individuals who engaged in physical distancing, using the tidy LPA package in R (Berlin et al., 2014; Rosenberg et al., 2018). Mean differences were tested across the latent profiles.

Four different models containing from 1 to 7 profiles were evaluated using the Bayesian Information Criterion (BIC) and Akaike Information Criterion (AIC) fit indices (Schwarz, 1978). Lower values for the AIC and BIC indicate better model fit and demonstrate the fewest parameters among a set of non-hierarchical models. The BIC is preferable in determining the best model fit over the other fit statistics (Nylund et al., 2007). Moreover, a bootstrap likelihood ratio test (BLRT) examined the statistical significance for model comparisons, by comparing the neighboring models as the number of classes decrease (McLachlan and Peel, 2000). A significant *p*-value for this test also indicates better model fit as profiles are added. To assess the certainty with which participants were classified into latent profiles, the entropy value was also considered, since it provides information regarding the accuracy of the models, with a range from 0 to 1. An entropy value of 0.80 or above is preferred and indicates an accuracy of >90% (Berlin et al., 2014). The best fitting model was chosen based on these criteria. See Table 2 for sample mean and standard deviations of the coping variables.

#### Multiple Linear Regression

Multiple linear regression models were used to explore the relationship between coping styles, personal wisdom, social support, self-transcendent wisdom, religiosity, spirituality, health, meaning in life, negative emotions, loneliness, alienation, and well-being. In particular, we are interested in investigating how these variables affected: (1) participants' retrospective pre-physical distancing well-being, and (2) the variance in reported current well-being (at the time of data collection) not explained by its pre-distancing level.

Descriptive statistics of the outcome and predictor variables are presented in Table 3. In preparation for multiple regression analyses, study variables were mean-centered by subtracting total sample mean from subscale or total scores. For variables where retrospective pre-physical distancing ratings were available (i.e., well-being, health, presence of and search for meaning in life, negative emotion, loneliness), unstandardized residuals were computed by regressing “during-physical distancing” scores on their respective pre-physical distancing values, to parse out the contribution of the baseline pre-physical distancing condition from change in performance during physical distancing and to mitigate multicollinearity (Table 4).

**Table 3:** Descriptive statistics of study variables (*N* = 614).

**Variable**	***M***	***SD***	**Range**
**Brief COPE**			
Self-distraction	3.50	1.63	0-6
Active coping	3.52	1.57	0-6
Denial	0.62	1.26	0-6
Substance use	0.92	1.56	0-6
Emotional support	2.91	1.73	0-6
Instrumental support	2.36	1.66	0-6
Behavioral disengagement	1.01	1.45	0-6
Venting	1.87	1.42	0-6
Positive reframing	3.15	1.75	0-6
Planning	3.51	1.73	0-6
Humor	2.10	1.86	0-6
Acceptance	4.42	1.41	0-6
Religion	1.81	2.15	0-6
Self-blame	1.17	1.51	0-6
**Coping profiles**^b^			
Religious coping	0.23	0.42	0-1
Substance Use	0.07	0.26	0-1
Average	0.61	0.49	0-1
Disengagement	0.08	0.28	0-1
Religiosity	3.40	3.65	0-10
Spirituality	4.56	3.63	0-10
Self-transcendence (ASTI)	2.63	0.54	1-4
Alienation^c^	2.32	0.59	1-4
Wisdom (Brief3DWS)	3.54	0.66	1-5
Social support (MSPSS)	5.42	1.23	1-7
Health (pre-physical distancing)	6.78	2.24	0-10
Health (current)^a^	6.61	2.30	0-10
Well-being (pre-physical distancing)	6.89	1.94	0.13-10
Well-being (current)^a^	6.47	2.06	0.13-10
Presence of meaning in life (pre-physical distancing)	4.57	1.64	1-7
Presence of meaning in life (current)^a^	4.23	1.54	1-7
Search of meaning in life (pre-physical distancing)	4.22	1.60	1-7
Search of meaning in life (current)^a^	4.20	1.63	1-7
Negative emotions (pre-physical distancing)	3.90	2.31	0-10
Negative emotion (current)^a^	4.21	2.48	0-10
Loneliness (pre-physical distancing)	4.20	3.22	0-10
Loneliness (current)^a^	4.66	3.25	0-10

a n = 613.

b A nominal variables based on the classifications from the LPA. Descriptive statistics should be read as the percentage of participants classified to each profile.

c *n = 608*.

**Table 4:** Predicting current levels of performance with retrospective pre-physical distancing levels.

**Current levels ON pre-physical distancing levels**	***B***	***S.E*.**	***Adjusted R^**2**^***	***F***
Well-being	0.84^***^	0.03	0.62	*F*_(1,611)_ = 1002.73^***^
Health	0.87^***^	0.02	0.71	*F*_(1,611)_ = 1513.79^***^
Negative emotion	0.76^***^	0.03	0.50	*F*_(1,611)_ = 604.53^***^
Loneliness	0.56^***^	0.03	0.31	*F*_(1,611)_ = 275.42^***^
Presence of meaning in life	0.69^***^	0.03	0.54	*F*_(1,611)_ = 716.38^***^
Search for meaning in life	0.74^***^	0.03	0.53	*F*_(1,611)_ = 689.41^***^

*** *p < 0.001*.

Nested robust multiple linear regressions were conducted in Stata/IC 16. Coping profiles obtained from the LPA were transformed into dummy variables, using the profile of participants who mainly used adaptive coping strategies as the reference group. Examinations of the correlation table (see Appendix A) and scatter plots suggested possible interaction effects between coping profiles and self-transcendent wisdom; therefore, a side-by-side comparison was made between models with and without the interaction terms. Additional *F*-tests were carried out to examine the statistical significance of the slopes of the predictor variable self-transcendent wisdom for people belonging to each coping profile. Paired-sample *t*-tests and one-way ANOVA analyses were conducted to explore possible group differences by nation, gender, educational level, marital status, religious affiliation, and annual household income in 2019 (See Appendix B for details). All models controlled for the main effects of nation, gender, educational level, marital status, religious affiliation, and household income prior to physical distancing (i.e., 2019).

#### Personal Projects Analysis

To elaborate on possible reasons for any changes in experience, we used personal projects analysis (Little, 1983) to examine how participants spent their time both before and after physical distancing measures went into effect. With the results of the regression analysis suggesting an important role of self-transcendent wisdom, we conducted an extreme cases analysis of the personal projects of the participants with the highest (*n* = 76) and lowest (*n* = 76) self-transcendent wisdom scores, ~25% of the total sample. Logistic regressions were employed to explore the effects of demographic characteristics on high-low self-transcendence group membership. The first and fourth authors, coding independently, content-coded participants' personal projects. The first author is a researcher in humanistic psychology with previous experience using the Personal Projects Analysis Workbook (Kim et al., 2020), while the fourth author has previous experience coding life narrative research. Personal projects were sorted into categories established by previous research in personal projects analysis (Little and Gee, 2007). Discrepancies were resolved *via* discussion between the authors.

## Results

### Latent Profile Analysis

**Figure 1:**
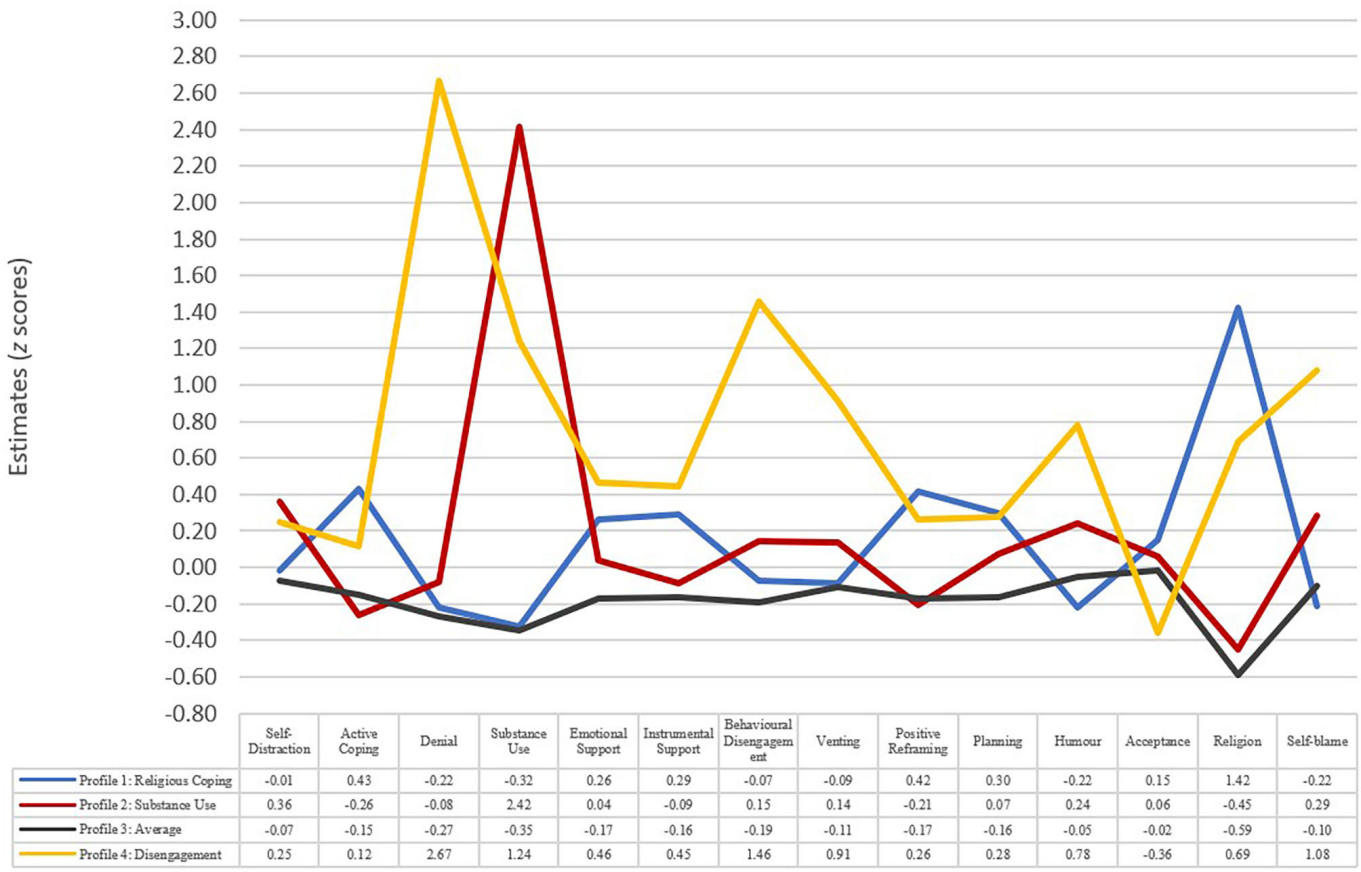
The four latent profiles of coping mechanisms among individuals who are physically distancing. Profile 1: religious coping (*n* = 144), Profile 2: substance use (*n* = 46), Profile 3: average (*n* = 373), Profile 4: disengagement (*n* = 51).

After comparing fit indices and entropy levels across all four models (see Table 5), a four-profile model was chosen to best classify coping strategies of participants who were physically distancing (see Figure 1 and Table 6). Each profile has been named after its most prominent form of coping, or, in the case of Profile 4, a core feature.

**Table 5:** Fit statistics for LPA (Model 3: equal variances and equal covariances).

**Classes**	**AIC**	**BIC**	**Entropy**	**Prob Min**	**Prob Max**	**%min**	**%max**	**BLRT *p*-value**
1	22332.82	22858.80	1.00	1.00	1.00	1.00	1.00	
2	21972.82	22565.10	0.97	0.98	1.00	0.15	0.85	0.01
3	21687.36	22345.94	0.94	0.95	0.98	0.10	0.65	0.01
4	**21500.75**	**22225.63**	**0.95**	**0.96**	**0.98**	**0.07**	**0.61**	**0.01**
5	21657.69	22448.87	0.79	0.78	0.97	0.10	0.34	0.94
6	21389.82	22247.30	0.81	0.77	1.00	0.06	0.31	0.01
7	21387.94	22311.72	0.84	0.80	0.98	0.07	0.24	0.15

*The selected model is bolded*.

**Table 6:** Mean differences between profiles by coping mechanisms.

	**Religious coping**			**Substance use**			**Average**			**Disengagement**		
	**(*****n*** **=** **144)**			**(*****n*** **=** **46)**			**(*****n*** **=** **373)**			**(*****n*** **=** **51)**		
	**Mean**	***SD***	***z***	**Mean**	***SD***	***Z***	**Mean**	***SD***	***z***	**Mean**	***SD***	***z***
Self-distraction	3.49	1.59	−0.014	4.09	1.55	0.364^*^	3.38	1.67	−0.074	3.90	1.36	0.246^*^
Active coping	4.20	1.32	0.433^**^	3.11	1.49	−0.264^*^	3.29	1.61	−0.151^*^	3.71	1.43	0.117
Denial	0.33	0.69	−0.222^**^	0.52	0.89	−0.082	0.28	0.67	−0.270^**^	3.98	1.22	2.667^**^
Substance Use	0.41	0.87	−0.323^**^	4.72	0.96	2.416^**^	0.38	0.78	−0.346^**^	2.86	1.51	1.238^**^
Emotional support	3.33	1.72	0.264^*^	2.98	1.90	0.039	2.62	1.70	−0.170^*^	3.75	1.29	0.462^**^
Instrumental support	2.84	1.68	0.292^*^	2.24	1.57	−0.087	2.10	1.62	−0.163^*^	3.10	1.55	0.446^*^
Behavioral disengagement	0.90	1.43	−0.072	1.24	1.58	0.146	0.73	1.17	−0.191^**^	3.16	1.53	1.460^**^
Venting	1.73	1.42	−0.086	2.07	1.32	0.135	1.72	1.32	−0.109	3.20	1.47	0.911^**^
Positive Reframing	3.88	1.72	0.415^**^	2.76	1.93	−0.208	2.85	1.68	−0.170^**^	3.61	1.48	0.260^*^
Planning	4.04	1.61	0.300^**^	3.65	1.69	0.072	3.22	1.76	−0.163^*^	4.02	1.35	0.277^*^
Humor	1.65	1.79	−0.221^*^	2.52	2.11	0.241	2.02	1.78	−0.052	3.59	1.54	0.781^**^
Acceptance	4.62	1.42	0.152	4.50	1.28	0.063	4.40	1.37	−0.017	3.92	1.64	−0.362^*^
Religion	4.88	1.09	1.422^**^	0.83	1.51	−0.451^**^	0.54	0.88	−0.588^**^	3.31	1.73	0.691^**^
Self-blame	0.82	1.30	−0.215^*^	1.63	1.58	0.286^*^	1.03	1.40	−0.101^*^	2.82	1.73	1.081^**^

z-standardized mean difference from sample mean.

* p < 0.05,

** *p < 0.001*.

Profile 1: Religious (23.5%) consisted of individuals who engaged in greater religious coping, z = 1.42, greater adaptive coping strategies (e.g., active coping, z = 0.43), and less maladaptive strategies (e.g., substance use, z = −0.32).

Profile 2: Substance Use (7.49%) consisted of individuals who engaged in greater maladaptive strategies (e.g., substance use, z = 2.42 and self-blame, z = 0.29) and less adaptive coping strategies (e.g., active coping, z = −0.26) and religious coping, z = −0.45.

Profile 3: Average (60.75%) consisted of participants who engaged in both adaptive and maladaptive strategies; they also engaged in very low levels of religious coping (z = −0.59).

Profile 4: Disengagement (8.31%) engaged in more behavioral disengagement, z = 1.46, substance use, z = 1.23, denial, z = 2.67, self-blame, z = 1.08, some adaptive strategies at higher levels (e.g., instrumental support, z = 0.45), and religious coping at higher levels, z = 0.69, but with less acceptance (z = −0.36).

These results suggest individuals engaged in both adaptive and maladaptive coping strategies. However, they also show that a small group of individuals were coping relatively well with the pandemic (23.5%). These profile memberships were used for subsequent analysis as the entropy level was high (0.95).

### Regression

Nested robust multiple linear regressions were used to predict both retrospectively reported pre-physical distancing well-being and current well-being not accounted for by pre-physical distancing levels. Regression models are presented in Table 7. Studentized residuals from models 2, 3, 4, 6, and 7 were normally distributed. While the removal of responses with outstanding studentized residual, leverage, or Cook's d slightly increased the amount of variance explained by the predictor variables, the models largely remained the same. Therefore, these responses have been kept in the regression analyses table.

**Table 7:** Predictive models of participants' reported levels of well-being (variables were mean-centered).

**Predictors**	**DV: retrospective pre-physical distancing well-being**				**DV: residualized current well-being**		
	**Model 1**	**Model 2**	**Model 3**	**Model 4**	**Model 5**	**Model 6**	**Model 7**
	***b***	***b***	***B***	***B***	***b***	***b***	***b***
	**(robust *SE*)**	**(robust *SE*)**	**(robust *SE*)**	**(robust *SE*)**	**(robust *SE*)**	**(robust *SE*)**	**(robust *SE*)**
Nation (Canada vs. USA)	0.13	−0.23^**^	−0.21^**^	−0.24^**^	−0.08	−0.11	−0.14
	(0.16)	(0.08)	(0.08)	(0.08)	(0.11)	(0.07)	(0.07)
Gender	−0.12	−0.26^**^	−0.23^**^	−0.25^**^	0.10	−0.00	−0.03
	(0.15)	(0.08)	(0.08)	(0.08)	(0.11)	(0.07)	(0.08)
Household income (2019)	0.24^***^	0.04	0.03	0.03	−0.04	−0.02	−0.01
	(0.04)	(0.02)	(0.02)	(0.02)	(0.03)	(0.02)	(0.02)
Religious affiliation	−0.18^***^	0.00	0.01	0.01	−0.03	0.03	0.02
	(0.03)	(0.02)	(0.02)	(0.02)	(0.02)	(0.02)	(0.02)
Highest education	0.12	−0.13^*^	−0.11	−0.11	0.11	0.01	0.01
	(0.11)	(0.06)	(0.06)	(0.06)	(0.08)	(0.05)	(0.05)
Marital status	0.44^**^	0.04	0.05	0.05	0.21^*^	0.06	0.05
	(0.14)	(0.06)	(0.06)	(0.06)	(0.09)	(0.05)	(0.05)
**Coping profiles**^**a**^							
Substance use		−0.02	0.02	0.03		0.10	0.12
		(0.18)	(0.17)	(0.18)		(0.16)	(0.17)
Average		−0.01	0.05	0.01		0.12	0.04
		(0.12)	(0.12)	(0.13)		(0.11)	(0.11)
Disengagement		0.63^**^	0.51^**^	0.19		0.38^*^	0.03
		(0.19)	(0.16)	(0.21)		(0.17)	(0.26)
Self–transcendence (ASTI)			0.53^***^	0.46^**^		0.49^***^	0.30^*^
			(0.10)	(0.16)		(0.09)	(0.14)
Substance Use*ASTI				0.22			0.48
				(0.27)			(0.24)
Average*ASTI				0.01			0.15
				(0.19)			(0.16)
Disengagement*ASTI				1.03^**^			1.19^*^
				(0.39)			(0.48)
Wisdom (Brief3DWS)		0.19^*^	0.22^**^	0.21^**^		0.07	0.06
		(0.08)	(0.07)	(0.07)		(0.07)	(0.07)
Social support (MSPSS)		0.40^***^	0.42^***^	0.41^***^		0.18^***^	0.18^***^
		(0.05)	(0.04)	(0.04)		(0.04)	(0.04)
Religiosity		0.02	0.02	0.02		0.04^*^	0.03
		(0.02)	(0.02)	(0.02)		(0.02)	(0.02)
Spirituality		−0.01	−0.02	−0.02		−0.01	−0.00
		(0.02)	(0.02)	(0.02)		(0.02)	(0.02)
Presence of meaning in life (pre-physical distancing)		0.48^***^	0.44^***^	0.45^***^		0.08^*^	0.09^**^
		(0.04)	(0.04)	(0.04)		(0.03)	(0.03)
Presence of meaning in life (current resid)			0.06	0.05		0.31^***^	0.30^***^
			(0.04)	(0.05)		(0.05)	(0.04)
Search of meaning in life (pre-physical distancing)		0.05^*^	0.03	0.03		−0.00	−0.00
		(0.03)	(0.03)	(0.03)		(0.02)	(0.02)
Search of meaning in life (current resid)			0.02	0.03		−0.02	−0.01
			(0.04)	(0.04)		(0.04)	(0.04)
Health (pre-physical distancing)		0.24^***^	0.22^***^	0.21^***^		0.04	0.04
		(0.02)	(0.02)	(0.02)		(0.02)	(0.02)
Health (current resid)			0.04	0.04		0.31^***^	0.29^***^
			(0.04)	(0.04)		(0.04)	(0.04)
Negative emotions (pre-physical distancing)		−0.08^**^	−0.07^*^	−0.07^**^		0.00	0.00
		(0.03)	(0.03)	(0.03)		(0.02)	(0.02)
Negative emotion (current resid)			−0.01	−0.01		−0.11^***^	−0.11^***^
			(0.03)	(0.03)		(0.03)	(0.03)
Loneliness (pre-physical distancing)		−0.05^**^	−0.05^**^	−0.06^**^		−0.01	−0.01
		(0.02)	(0.02)	(0.02)		(0.01)	(0.01)
Loneliness (current resid)			−0.03	−0.03		−0.04^*^	−0.04^*^
			(0.02)	(0.02)		(0.02)	(0.02)
Alienation			0.01	−0.02		−0.38^***^	−0.41^***^
			(0.09)	(0.09)		(0.10)	(0.10)
Well-being (pre-physical distancing)						−0.28^***^	−0.30^***^
						(0.04)	(0.04)
Well-being (current resid)			−0.33^***^	−0.34^***^			
			(0.05)	(0.05)			
Intercept	−1.27^**^	0.69^*^	0.53	0.65^*^	−0.35	−0.09	0.07
	(0.45)	(0.30)	(0.29)	(0.29)	(0.37)	(0.26)	(0.26)
*R^2^*	0.15	0.78	0.81	0.81	0.02	0.61	0.62
*N*	604	604	604	604	604	604	604

a Dummy variables of coping styles were created with “using mainly adaptive coping strategies” as the reference group.

* p < 0.05,

** p < 0.01,

*** *p < 0.001*.

Models 1 and 5 were baseline models examining the main effects of nation, gender, household income in 2019, religious affiliation, educational level, and marital status on participants' pre-physical distancing well-being as well as the amount of variance in current well-being not explained by its pre-physical distancing level. Both models were significant, *F*_*m*1__(6,597)_ = 17.79, *p* < 0.001, *F*_*m*5__(6,597)_ = 2.50, *p* = *0.0*2, but only about 15 and 2% of the variance in pre-physical distancing well-being and the unstandardized residuals of current well-being respectively were accounted for.

Models 2 and 3 aimed to predict pre-physical distancing well-being using slightly different approaches. In Model 2, we predicted pre-physical distancing well-being using pre-physical distancing levels of negative emotion, loneliness, health, presence of and search for meaning in life, as well as constructs hypothesized to be largely stable over time (i.e., coping profiles, wisdom, social support, religiosity, and spirituality). However, since our pre-physical distancing well-being was a retrospective measure that might be significantly affected by current levels of performance, Model 3 examined the effects of current levels of negative emotion, loneliness, health, presence of and search for meaning in life, represented by unstandardized residuals, as well as difference measures of self-transcendence and alienation measured by ASTI.

As shown in Table 7, both Model 2 and 3 significantly predicted pre-physical distancing well-being, *F*_*m*2__(18,585)_ = 106.73, *p* < 0.001, *F*_*m*3__(26,577)_ = 85.98, *p* < 0.001. Model 2 explained significantly more variance than the baseline model, *F*_*change*(2−1)__(12,585)_ = 134.92, *p* < 0.001, and Model 3 explained significantly more variance than Model 2, *F*_*change*(3−2)__(8,577)_ = 10.89, *p* < 0.001, but only for an additional 3.16% of the variance. Furthermore, among the additional predictors added in Model 3, only ASTI and the residualized current well-being turned out to be significant. The significance main effect of ASTI may be explained by the fact that it was a change score, thus had the pre-physical distancing level of self-transcendent wisdom already embedded within it. The unstandardized residuals of current well-being were negatively associated with retrospective pre-physical distancing well-being. Overall, after controlling for the other variables in Models 2 and 3, pre-physical distancing well-being was positively associated with personal wisdom, social support, and retrospective pre-physical distancing levels of presence of meaning in life and health, and was negatively associated with pre-physical distancing negative emotions and loneliness.

**Figure 2:**
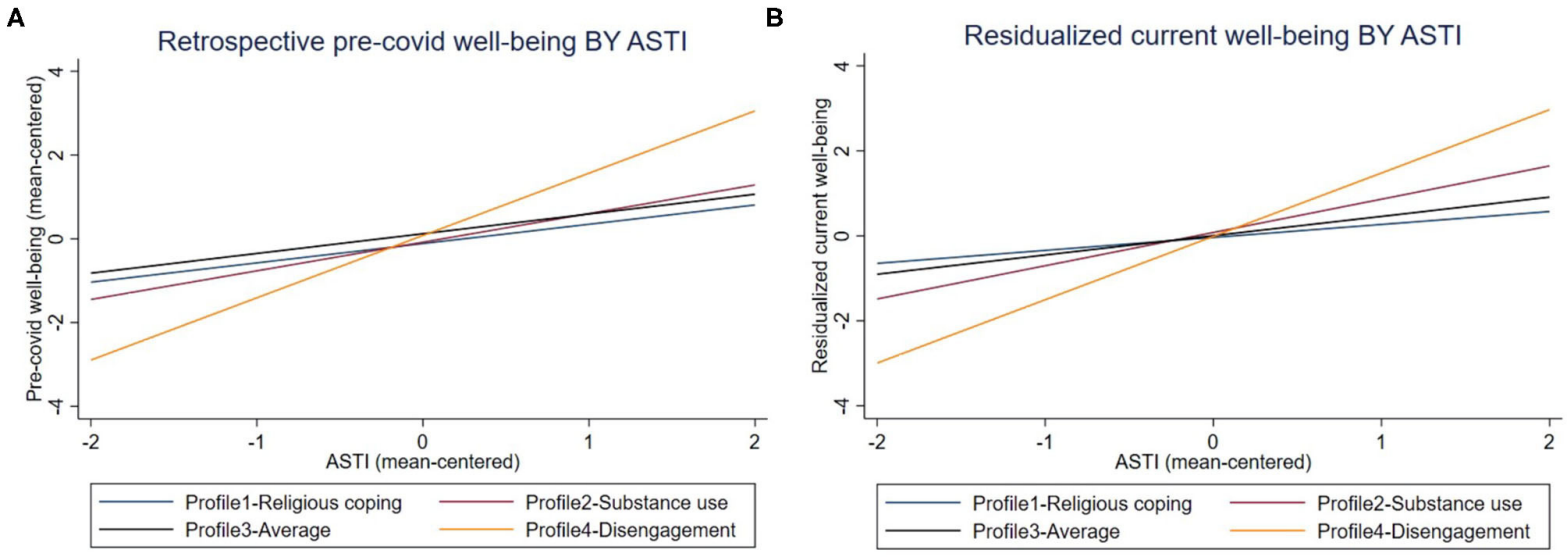
The effects of self-transcendent wisdom on self-reported levels of well-being for people belonging to different coping profiles (variables were mean-centered). For illustrative purposes, **(A,B)** were created for an individual that fell into the median categories for all demographic variables (Canadian, male, Christian, married, or living with a partner, had a Bachelor's degree or equivalent, and lived in a household the annual income of which was between C$40–60k in 2019) and scored the mean values for all continuous predictors except for ASTI (i.e., unstandardized residuals = 0).

Before entering the interaction terms between coping profiles and ASTI, controlling for all other variables in the models, belonging to coping Profile 4 “Disengagement” was associated with higher pre-physical distancing well-being. Model 4 explored the interaction terms, *F*_*m*4__(29,574)_ = 78.20, *p* < 0.001. While the additional explanation power of Model 4 over Model 3 was only 0.34%, this change was significant, *F*_*change*(4−3)__(3,574)_ = 2.93, *p* = 0.03. Model 4 revealed a significant interaction effect between ASTI and Disengagement membership (see Figure 2A). *F*-tests were conducted to see (1) whether the slopes of the regression lines in Figure 2A were significantly different from zero, and (2) whether the slopes of these lines were significantly different from one another. Results are presented in Table 8. The slopes of ASTI for all coping profiles were significant, and significantly different from one another as demonstrated by the significant *R*^2^ change between Model 3 and 4. The slope of the regression line for people with a Disengagement profile was significantly steeper than those with a Religious coping profile or used an average level of all coping strategies (Profile 3).

**Table 8:** F-tests for significant non-zero slopes and significant differences between the slopes of pre-physical distancing well-being on ASTI by each coping profile.

**Comparisons**	**Model 4**	**Model 7**
**Significantly different from zero**		
Religious coping	*b* = 0.46^**^^a^	*b* = 0.31^*^^a^
Substance use	*b* = 0.68, *F*_(1,574)_ = 8.66^**^	*b* = 0.78, *F*_(1,574)_ = 13.62^***^
Average	*b* = 0.47, *F*_(1,574)_ = 17.59^***^	*b* = 0.45, *F*_(1,574)_ = 18.52^***^
Disengagement	*b* = 1.49, *F*_(1,574)_ = 16.47^***^	*b* = 1.49, *F*_(1,574)_ = 10.35^**^
**Significantly different from one another**		
Religious coping vs. Substance use	*F*_(1,574)_ = 0.67	*F*_(1,574)_ = 3.86^*^
Religious coping vs. Disengagement	*F*_(1,574)_ = 6.87^**^	*F*_(1,574)_ = 6.03^*^
Religious coping vs. Average	*F*_(1,574)_ = 0.00	*F*_(1,574)_ = 0.86
Substance Use vs. Disengagement	*F*_(1,574)_ = 3.83	*F*_(1,574)_ = 1.96
Substance Use vs. Average	*F*_(1,574)_ = 0.78	*F*_(1,574)_ = 2.19
Disengagement vs. Average	*F*_(1,574)_ = 8.23^**^	*F*_(1,574)_ = 4.80^*^

a This is the coefficient of ASTI in Model 4 or 7.

* p < 0.05,

** p < 0.01,

*** *p < 0.001*.

Model 6 aimed to improve on the baseline Model 5 (discussed above) by adding pre-physical distancing and residualized current levels of negative emotion, loneliness, presence of and search for meaning in life, and health, as well as coping profiles, wisdom, social support, religiosity, spirituality, self-transcendent wisdom, alienation, and pre-physical distancing well-being, in the prediction of residualized current well-being. Model 6 explained 61.47% of the variance in the unstandardized residuals of current well-being, *F*_*m*6__(26,577)_ = 25.01, *p* < 0.001, representing a significant *R*^2^ change compared to Model 5, *F*_*change*(6−5)__(20,577)_ = 30.47, *p* < 0.001. Model 7 further added the interaction terms between coping profiles and ASTI and explained 62.47% of the variance in the dependent variable, *F*_*m*7__(29,574)_ = 23.36, *p* < 0.001. While quite small, this *R*^2^ change was also significant, *F*_*change*(7−6)__(3,574)_ = 2.91, *p* = 0.03.

Predicting variance in current well-being not accounted for by pre-physical distancing levels, after controlling for other variables, we observed significant positive main effects of social support, pre-physical distancing and residualized current presence of meaning in life, residualized current health, and ASTI, as well as significant negative main effects from residualized current negative emotion and loneliness, alienation, and retrospective pre-physical distancing well-being. The effects of the interaction terms are illustrated in Figure 2B, and the results of *F*-tests are shown in Table 8. The slopes for ASTI were significant for all coping profiles, and significantly different from one another as demonstrated by the significant *R*^2^ change between Model 6 and 7. The slope of the regression line for people with a Disengagement profile was significantly steeper than those with a Religious coping profile or an average level of all coping strategies (Profile 3). The slope of the regression line for people with a Substance Use profile was significantly steeper than with a Religious coping profile.

### Extreme Case Analysis of High and Low Transcenders

Since self-transcendent wisdom plays such an important role in maintaining and improving well-being, we decided to further examine the individuals with particularly high and low self-reported transcendence. Using logistic regressions, we explored the effects of demographic characteristics (i.e., country, age, gender, annual household income in 2019, religious affiliation, marital status, and highest level of education) on whether one fell into the top and bottom 10% based on their scores on ASTI (*n* = 76 participants, per group).

As shown in Table 9, ASTI extreme group membership is significantly associated with religious affiliation. In particular, whether or not one adjusts for the effects of other demographic characteristics, the odds of being in the high-ASTI group for Christians were 2.94, those for Atheists were 0.30, those for Agnostics were 0.36, and were 1.30 for all others. Due to the more restricted sample size (*n* = 152), we were not able to compare the relative odds of being a Muslim, Buddhist, or Hindu.

**Table 9:** Predicting memberships in the high and low ASTI groups by demographics.

**Predictors^***a***^**	**Model 1**	**Model 2**	**Model 3**	**Model 4**
	**Odds ratio**	**Odds ratio**	**Odds ratio**	**Odds ratio**
	**(S.E.)**	**(S.E.)**	**(S.E.)**	**(S.E.)**
**USA**	1.01			
	(0.46)			
**Age**^**c**^	0.98			
	(0.02)			
**Female**	0.49			
	(0.20)			
**Household income (2019)**^***c***^	1.00			
	(0.11)			
**Religious affiliation**^***d***^				
Atheist	0.09^***^	0.10^***^		
	(0.05)	(0.05)		
Agnostic	0.11^***^	0.12^***^		
	(0.06)	(0.06)		
Other	0.48	0.44		
	(0.28)	(0.23)		
**Highest education**				
High school and below	0.48		0.45^*^	
	(0.21)		(0.17)	
Graduate degree and equivalent	1.08		0.12	
	(0.63)		(0.55)	
**Marital status**^***b***^				
Never married	0.51			0.47^*^
	(0.23)			(0.16)
Divorced/separated/widowed	3.88			1.25
	(3.25)			(0.84)
**Intercept**	6.40^**^	2.94^***^	1.29	1.40
	(3.41)	(0.85)	(0.29)	(0.33)
***χ^2^***	46.82^***^	34.01^***^	5.47	5.73
**Pseudo** ***R**^**2**^*	0.22	0.16	0.03	0.03
***N***	151	151	151	151

a Median categories were chosen as the reference group.

b “Divorced/separated” and “Widowed” were combined because the cases belonging to “widowed” was only 6.

c mean-centered.

d “Buddhist,” “Muslim,” and “Hindu” were coded into “other” due to very small count (n_Buddhist_ = 1, n_Hindu_ = 3, n_Muslim_ = 5).

* p < 0.05,

** p < 0.01,

*** *p < 0.001*.

### Qualitative Results

**Figure 3:**
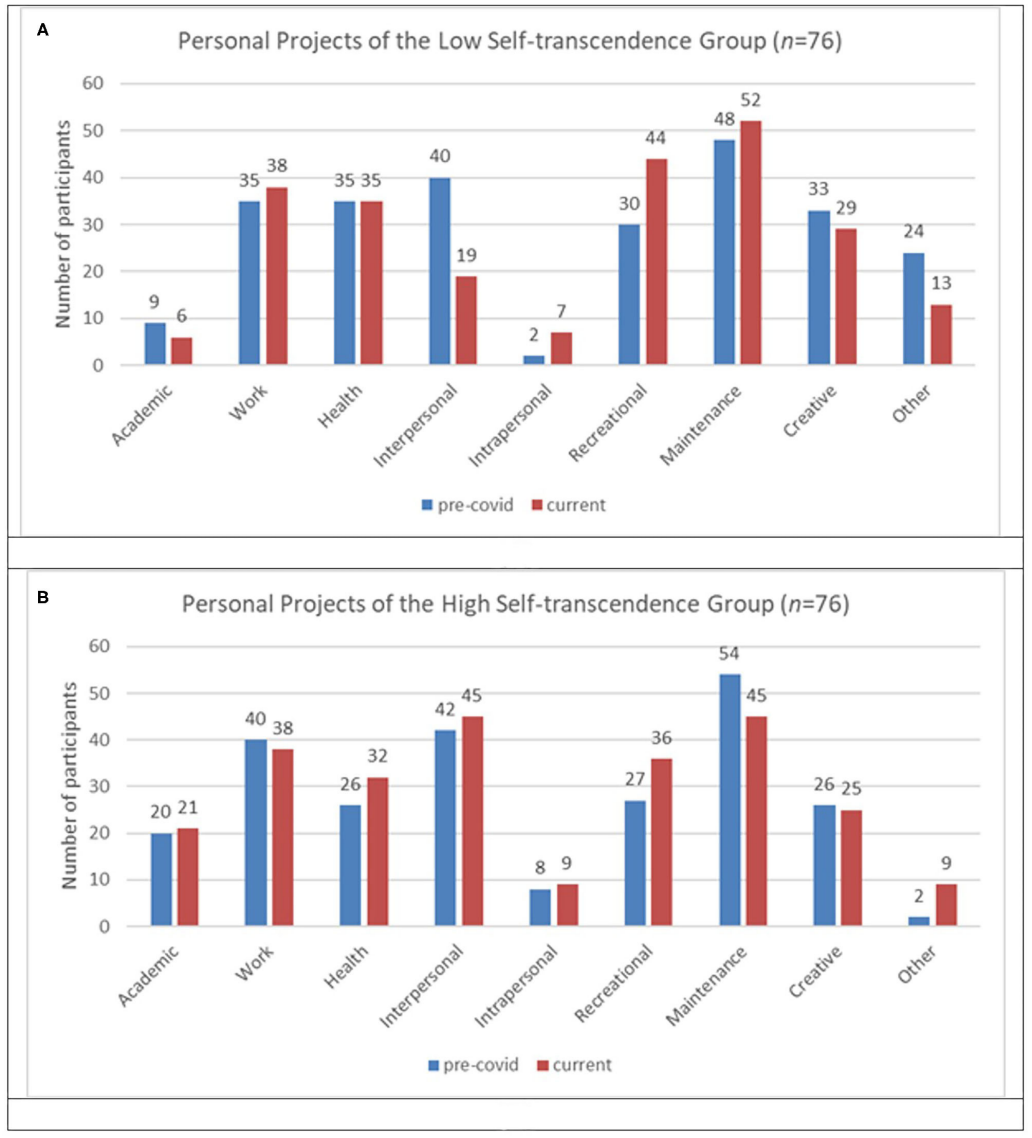
**(A)** Personal projects reported by participants low in self-transcendent wisdom and **(B)** high in self-transcendent wisdom.

Each personal project reported was categorized as one of nine possible types: Academic, Work, Health, Recreational, Interpersonal, Intrapersonal, Maintenance, Creative, and Other. All categories come from the established work on personal projects analysis (Little and Gee, 2007), except for “Creative” projects, added to classify projects that were neither academic, work, or recreational. “Other” projects did not fit cleanly within any existing category, nor align with other idiosyncratic items to suggest a new category, and were excluded from the present analysis. To highlight the prevalence the particular types of projects among participants before and during physical distancing, Figure 3 and Tables 10, 11 report the number of participants with high or low self-transcendent wisdom who reported at least one project of the listed type. In what follows, we present only those categories that differ substantially between groups.

**Table 10:** Examples of pre and current project for the low self-transcendence group.

**Project category**	**Pre-physical distancing project examples**	**Current project examples**
Academic	Study Math; Finish dissertation; Study for a higher certification; Complete introductory course for degree.	Study; Finish dissertation; Online courses; Going back to school; School; Working on my graduate research.
Work	Trying to get better at my job by doing lots of unpaid overtime; Find a better career; Go to work; Running a side business.	Working online; I work from home; Doing online gigs like MTurk; Making money on the side to make up for lost business income.
Health	Exercise regularly; Lose weight; Working out; Eating clean; Following up on doctor visits.	Keeping hands clean; Lose weight; Fitness goals; Yoga; Exercise at home; Lifting weights/getting bigger.
Recreational	Play games; going to movies; Finish reading a book I had started; Watch TV; Watch movies.	Play Sims; Building an island in Animal Crossing; Playing video games; Reading more; Watching Netflix.
Maintenance	Cleaning the house; Grocery shopping; Yard work; Cooking dinner; Mowing the lawn.	Home repairs; Give my dog a bath; Get a haircut; Obtain stock of cleaning products; Keeping the house clean; Cleaning out the garage.
Interpersonal	Finding romance; Write brother; Taking care of parents; Making online friends; Hanging out with friends; Visiting family; Dating.	Video chatting with friends and family; Playing games with friends; Homeschooling kids to prepare for the next grade; Raise child
Intrapersonal	Strengthening my spirituality; Continuing my mental health therapy.	Try to be a nicer person; Get better control of my anger; Keeping my spirit up; Trying to appreciate the time off work.
Creative	Learning guitar; Painting; Personal animation projects; Woodworking; Work on my classical singing.	Baking; Redecorating my room; Baking bread; Cocktail making; Diamond painting; Work on editing my novel.

*Note: The pre-physical distancing and current lists are not from the same participants*.

**Table 11:** Examples of pre and current project for the high self-transcendence group.

**Project category**	**Pre-physical distancing project examples**	**Current project examples**
Academic	Finish my degree; Pursuing my Masters education; Pursuing my PhD; Upgrading my math for future college courses.	Studying for school; Online professional course; Pursuing my Masters; Take online courses; Studying for a professional exam.
Work	Setting up an online business; Working a new business idea; Work. Complete work documentation; Increase income streams.	Going to work; I have been getting extra shifts; Online work; Earning money working online; Research about work; New business.
Health	Dental check-up appointment; Going to the gym daily to improve my health; Fitness; Hiking.	I am eating healthier; Increase stamina; Starting a new workout plan; Keeping myself busy and active (Running through the stairs, using treadmill).
Recreational	Reading; Watching TV and movies; Reading new books; Read more books; Movie and opera visit; Catch up on TV shows.	Read more; I am watching a TV series with my wife every night; Reading books; Read some classic books; Games; Catch up on favorite TV shows.
Maintenance	Cleaning out the shed; Work on car; Clean the house; Cooking; Buying groceries.	Working on house repair; Work on my car; Yard work; Cleaning up garage; Paint the inside of my house.
Interpersonal	Helping my daughters with basketball; Teach my son how to swim; Walking with friends daily; Go visit my dad; Taking my son to the park for socialization; Enjoying online game with friends.	I have had deep conversations with my family; Rekindling familial relationships; Generally getting to know people better from a virtual distance; Making deeper connections with people.
Intrapersonal	Taking better care of myself; Connecting more to my faith; Read a self-help book; Stay in touch with my spiritual side.	Trying to be as happy as possible; Thinking what it is to be satisfied and content in life; Meditating every day to relieve stress; Improving self-care.
Creative	Take photos; Rock painting; Knitting a scarf; Paint a picture; Practicing my piano; Write a book; Do some craft projects.	Make art; Practice playing the piano; Scrapbooking; Drawing; Writing my own novel; Card making; Canvas painting; Learning a new language.

*Note: The pre-physical distancing and current lists are not from the same participants*.

#### Recreational Projects

Both the high and low self-transcendent wisdom group participants placed greater emphasis on recreational projects at the time of data collection than they did reflecting on their projects prior to physical distancing, as seen in Figure 3. The low-transcendence group emphasized recreational pursuits that appeared more escapist in nature (e.g., playing video games, “binge” watching television series), whereas the high self-transcendent wisdom group emphasized reading books—often phrased as “catching up” on a pre-existing reading list. Where the high self-transcendent wisdom group also reported watching shows or playing games, it was often in the context of watching with family, or playing online games with friends. With such an emphasis, such projects were instead counted as interpersonal projects, reported below. Absent any mention of friends or family being involved in electronic entertainment, we theorize that playing video games and watching television series allowed participants to mimic missing social relationships through immersion in narratives, as well as being an accessible and understandable form of escapism (Hilgard et al., 2013; Blasi et al., 2019). While there is likely also an escapist element in reading projects, reading suggests exercising the mind and the imagination, rather than turning to more sensory distractions for fun. The emphasis on more sensory, concrete, or immediate projects is a recurring theme amongst projects of participants with the lowest levels of self-transcendent wisdom.

#### Interpersonal Projects

Participants with high self-transcendent wisdom placed greater emphasis on projects involving deepening interactions with other people, especially family. Prior projects involving family are maintained, or participants mention transitioning toward making their current projects more focused on spending time with their family, friends, or significant others. Participants with low self-transcendent wisdom had difficulty maintaining their interpersonal projects across pre-physical distancing and current lists: For example, dating and spending time with friends dropped off their current list. We can particularly see this with projects involving electronic media. For participants with high self-transcendent wisdom, projects involving watching television or playing videos games emphasize sharing an experience, and are therefore coded as “Interpersonal” projects. Those with higher levels of self-transcendent wisdom appeared to prioritize deepening the connections they have, while those with lower reported levels of self-transcendent wisdom appear to retreat from others, engaging with projects that are accomplished alone, or are immediately salient.

#### Intrapersonal Projects

Intrapersonal projects cover activities that attempt to change or develop oneself (e.g., active self-care, personal growth, and spirituality). While participants with high self-transcendent wisdom reported more intrapersonal projects prior to physical distancing than participants with low self-transcendent wisdom, we see a spike in these projects among latter group during physical distancing that focus on managing negative emotions, particularly anger. Given the association between self-transcendent wisdom and feelings of interconnectedness and personal growth, it makes sense that the projects of those with higher levels of self-transcendent involve identifying ways to feel more content, happy and well, through e.g., meditation and self-care, during a global pandemic that generates an unprecedent level of anxiety and stress about the future. The projects of those low in self-transcendent wisdom, on the other hand, again, emphasize something immediate and visceral; in this case, anger management.

#### Maintenance

Across both groups of participants, a qualitatively noticeable difference was noted for participants with low, as compared to high, self-transcendent wisdom, toward very basic forms of survival projects during physical distancing. Projects such as *grocery shopping, cooking*, and *cleaning* were far more central for participants with low self-transcendent wisdom, particularly in comparison with the projects they listed having prior to physical distancing. As was the case with “Recreational” projects, participants with high self-transcendent wisdom emphasized interpersonal interactions even in their maintenance projects. The most common example of this was *cooking with family*. Rather than just mentioning the project of cooking, they specifically noted that were doing the task with family. This emphasis on spending time with others during what would otherwise be routine task moved such projects to the “Interpersonal” projects section.

#### Creative Projects

We created the category of “Creative” projects as distinct from “Academic,” “Work,” and “Recreational” projects in order to capture artistic projects, musical projects, or in some cases, learning languages, that lacked an emphasis on formal schooling, earning money, or hedonic fun. Creative projects instead emphasized making things or mastering skills. Prior to physical distancing, participants in both groups reported similar projects. The most common of these projects were what one would traditionally categorize as creative pursuits, such as painting, writing, or playing an instrument. During physical distancing, however, projects began to diverge. For low transcendence participants, even creative projects retain some element of a utilitarian goal: preparing food with a new recipe, for instance, or redecorating personal spaces. Creative projects among high-transcendence participants, on the other hand, tended to emphasize creation for creation's sake, with more emphasis on languages, fine arts, crafts, or even puzzles. Once again, participants with lower self-transcendent wisdom focused on concrete, tangible projects that often have a clear goal or result, while participants with higher self-transcendent wisdom focused on projects that appear more open-ended, abstract, and typically involve others.

Overall, by examining the participants with the lowest and highest self-transcendent wisdom we see a pattern of change in pre-pandemic to current personal project lists. Examples of personal project responses that display a pattern of transformation are displayed in Table 12. The first and third example are participants with high self-transcendent wisdom, whereas the second example is a participant with low self-transcendent wisdom. Participants with high self-transcendent wisdom appeared to be more successful in staying connected with other people and larger causes. They often found new ways to spend their energy, either by connecting at a more local level or, in the case of one memorable participant displayed in Table 12, radically scaling up their circle of concerns to new heights of political activism. Participants with low self-transcendent wisdom, by contrast, appeared to withdraw rather than venture outwards. While some managed to adjust to a world in which connections must be either immediately local or radically global, overall the projects of the lowest scoring participants seem to have been, for lack of a better word, crushed.

**Table 12:** Exemplar participants pre-current personal project lists.

**Pre-pandemic personal projects**	**Current personal projects**
Trying to earn enough on mTurk to keep our family afloat; Trying to help my husband get into an alternate teaching certification program; Trying to keep my blood sugar at a reasonable level; Trying to lose weight; Trying to save enough money to bring our dog to the vet.	Starting our self-sufficient farm (growing our own vegetables, washing clothes by hand composting, etc.); working more on mTurk to be able to build our chicken coop; Fixing things in our home that have needed to be fixed for a long time; Using social media to encourage political and social change (urging Congress to pass UBI, Medicare4All, police restructuring, etc.); Writing government officials to express my opinions and urge change.
Learning guitar; Learning to drive; Visiting friends; Saving money; Learning music theory.	Learning guitar; Learning music theory; Watching movies with friends online; Playing online games with friends; Getting in better shape.
I was concentrating on my health; I was trying to be financially stable; I was invested in advancing my career; Being content with life; Having a happy married life.	Personal health; Thinking about all those loved ones and friends and family; Making deeper connections with people; Thinking about career change; Thinking of what it is to be satisfied and content in life; Having a fun and happy married life.

## Discussion

The analyses described above focused on determining predictors of two main variables: (1) retrospective perceptions of well-being before the beginning of physical distancing, and (2) the residuals between participants' retrospective well-being and participants' perceived well-being since the enactment of physical distancing measures, which we used to provide an estimate of change in perceived well-being since physical distancing began. Before we move to a general discussion, it is worth noting our overall approach to the interpretation of these data. Due to the retrospective nature of the data collection, we interpret these data used as representing idiographic participant self-understanding of change over time, as it seems to them at the time of data collection, rather than “objective” differences between present and past states of being. While psychology has historically emphasized nomothetic analyses of experience (Munsterberg, 1899), idiographic approaches to data championed by Allport (1942) or more recently Lundh (2015) are particularly applicable to the study of events which are, incontestably, historical in nature, like the present pandemic. Indeed, computer-assisted textual analysis has revealed narrative patterns consistent with the quantitative and qualitative findings from our study, namely, that one is either experiencing the current pandemic as a time for vital re-evaluation of priorities, or as a fight for survival (Venuleo et al., 2020).

In terms of quantitative findings, controlling for all other variables in the model, we observed a greater association between nationality, gender, personal wisdom, disengagement coping profile, retrospective health, loneliness, negative emotions, and meaning in life and participants' retrospective well-being than for other factors. Residualized current perceived health, negative emotions, loneliness, and alienation demonstrated the greatest association with residualized current well-being, again controlling for all other variables. The interaction between Disengaged coping and self-transcendent wisdom, as well as self-transcendent wisdom on its own, was strongly associated with both retrospective and residual well-being, as was retrospective meaning in life and social support. Controlling for all other variables, residualized current well-being is negatively associated retrospective pre-physical distanced well-being.

Many of these results are in line with past findings, both during and prior to the pandemic. For instance, personal wisdom (Grossmann et al., 2013; Ardelt, 2019), health (Aneshensel et al., 1984; Hayes and Ross, 1986; Hsieh and Waite, 2019; Park and Adler, 2003), and social support (Turner, 1981; Thoits, 1985; Portero and Oliva, 2007; Hyde et al., 2011) are known to be predictive of well-being. Personal wisdom in particular has been previously found to be a protective factor for psychological well-being during the COVID-19 pandemic (Pellerin and Raufaste, 2020), though it does not appear to impact residualized current well-being in the present sample. The coping responses from our participants also align with those who have experienced similar outbreaks (Chew et al., 2020; Rajkumar, 2020). Although some strategies (e.g., distraction) may be deemed maladaptive, evidence suggests their short-term efficacy in dealing with uncontrollable situations (Janson and Rohleder, 2017), explaining the existence of the Disengaged coping profile. While recent research by Park et al. (2020) also found evidence that demographic factors (e.g., age and socio-economic status) may predict differential coping responses with the pandemic, only nationality and gender appear to play a role in the models presented here. Of the two, Canada has had significantly fewer cases of COVID-19 relative to its population than the United States, as well as more centralized support from the federal government, while the pandemic is well-known to have had a disproportionate impact on women relative to men (Alon et al., 2020). In summary, there do not appear to be any surprises in predictors of current well-being.

In line with previous findings that strong interpersonal relationships predict more positive experiences of solitude (Pauly et al., 2018), our qualitative analyses found that participants with high self-transcendent wisdom strongly emphasized how their projects during times of physical distancing helped connect them to friends, family, and community. Previous research examining the presence of meaning in life have consistently found that meaning relates to *connectedness*, whether through existential mattering (Costin and Vignoles, 2020), relationships of mutual care and trust (Wong, 2020), or an overall sense of coherence (Park, 2010). Good relationships with others are also consistently placed among the most important aspects of what it means to live a good life the world over (Tafarodi et al., 2012; Bonn and Tafarodi, 2013). As such, the finding that the participants who are relatively thriving during periods of physical distancing have managed to center and maintain these relationships is consistent with several models of a good, meaningful life. The finding that participants with low self-transcendent wisdom have found it apparently difficult to maintain such relationships and seem to be slipping into escapism and self-distraction is consonant with the narratives of “global crisis” and “surviving a war” identified by Venuleo et al. (2020) as most frequent amongst people experiencing greater instability during lockdown measures in Italy. Combined with our quantitative findings that loneliness and alienation demonstrated negative associations with perceived well-being, such findings further evidence for the importance of strong, resilient relationships to weathering misfortune, particularly on misfortune on such a magnified scale.

That participants with the highest self-transcendent wisdom were more likely to be religious is an interesting finding, and one for which the framework of existential positive psychology provides some context. While it might be too strong a claim that religion itself is necessary, Wong's (2020) model of existential meaning emphasizes the importance of faith and concern for greater things in cultivating meaning, which religious participants could be reasonably expected to experience more strongly than non-religious participants. Religion is also known to have strong anxiolytic effects (Kay et al., 2010; Newton and McIntosh, 2010). Interestingly, self-transcendent wisdom, otherwise an important factor in predicting change in well-being, was had a reduced impact for participants with a Religious coping profile.

Self-transcendence is defined as decreased egoic self-salience and increased feelings of connectedness to something larger than oneself (Kitson et al., 2020). A sense of connectedness can provide an anchor point for coping (Frydenberg and Lewis, 2009), and strengthening that sense of connectedness underlies both self-transcendence (Kitson et al., 2020) and cultivation of meaning in life (George and Park, 2016; Costin and Vignoles, 2020). In developmental research, self-transcendence is related to—and perhaps a precondition for— developing wisdom (Aldwin et al., 2019), with the Adult Self-Transcendence Inventory being designed to evaluate such self-transcendent wisdom. Based on our findings, it appears that self-transcendent wisdom contributes to current well-being over and above coping style and predicts change in well-being for people using all coping styles, except for those using mainly Religious coping.

That self-transcendent wisdom seemed to have a stronger contribution to variance in well-being over coping for Profiles 2 (Substance Use) and 4 (Disengagement) than for Profiles 1 (Religious) and 3 (Average) is somewhat surprising. Perhaps wiser people have a deeper understanding of negative emotions as not necessarily bad or maladaptive; used appropriately, they can help signal the need to engage adaptive strategies for psychological safety or strategies that can generate psychological growth (Webster, in press). Likewise, even avoidance coping may be adaptive in certain clinical contexts [see Hofmann and Hay (2018)].

A decreased acceptance of ongoing negative events might also have played a role. Previous research in mindfulness has found that the “non-judgmental acceptance” component of mindfulness is negatively associated with both intuition (Remmers et al., 2014) and wise reasoning (Kim et al., 2020), suggesting that acceptance may circumvent the exploratory processing needed to achieve greater well-being (Weststrate, 2019). Pellerin and Raufaste (2020) similarly find that in the current pandemic climate, peaceful disengagement actually predicts a decline in well-being over time. Wiser individuals are particularly good at managing the sense of uncertainty and uncontrollability that necessarily comes with adverse experiences (Glück et al., 2018; Glück, in press). According to Glück et al.'s (2018) MORE model, wise individuals can sustain a higher level of negative emotions when trying to understand the complexity of a situation. Therefore, their use of different types of coping strategies can help maximize their well-being (Glück, in press).

However, for participants who already had adaptive coping mechanisms, their existing strategies probably accounted for most of the added benefit of self-transcendent wisdom, although self-transcendent wisdom still played a role in predicting their well-being. Coping strategies appear to be important for well-being in daily life (Ben-Zur, 2009), as well as for helping to maintain a baseline functioning during stressful events (Park and Adler, 2003). However, self-transcendent wisdom is the more robust indicator, when comparing coping and self-transcendence as indicators of well-being (McCarthy et al., 2013). Additionally, the use of Religious coping may overlap sufficiently with self-transcendent wisdom in motivating attention to concerns beyond the self so as to reduce any apparent impact.

Interestingly, self-transcendence was not a significant predictor of well-being in a previous study of protective factors during the COVID-19 pandemic. Pellerin and Raufaste (2020) found that self-transcendence was only related to well-being when all other potential psychological resources (i.e., personal wisdom, self-efficacy, optimism, hope, and gratitude) were not controlled for; thus, they consider self-transcendence a meta-resource that affords the development of other resources, or perhaps this reflects a floor effect: an individual may need higher self-transcendence in order to see a benefit. Our findings suggest that it is not greater self-transcendence, but rather a more holistic approach to it that is needed to see results. While Pellerin and Raufaste (2020) used only the self-transcendence ASTI subscale in their research, to specifically target self-transcendence as an independent variable, our study used all five subscales to measure self-transcendent wisdom in its full spectrum. Our findings also suggest that self-transcendent wisdom may make the biggest contribution when an individual lacks healthy, adaptive coping strategies. Participants with adaptive coping skills could simply apply what they already know to the present situation. Without such skills, however, self-transcendent wisdom may make the difference between a traumatic experience and a transformative one.

In the philosophical literature on self-transformation, a distinction is made between self-transformation and self-cultivation (Callard, 2018). Unlike self-transformation, self-cultivation involves no radical shift in values, but instead refines their ability to apply existing values. After self-cultivation, one is simply a more effective version of the same person they were before. Self-transformation, on the other hand, involves a radical shift in values (Paul, 2014). Sometimes, transformation is a consequence of unexpected situations, like unplanned parenthood. At other times, self-transformation is an active pursuit, as for spiritual aspirants, or in the best traditions of liberal education (Callard, 2017). In both cases, a person may be unrecognizable to their past self after self-transformation; their priorities, values, and approach to life may come to be entirely different than those they held before. In our current sample, participants with an adaptive coping profile seem to be engaged in self-cultivation —using and refining what they already know to be good for them—while participants with other coping profiles seek self-transformation.

Although the pandemic has severely affected every area of public life, it has also deepened some individuals' connections to others and complexified their worldview, which could potentially allow post-traumatic growth (PTG), defined as positive psychological change following trauma that results in a greater appreciation for life, increased personal strength, spiritual growth, more meaningful relationships, and the recognition of new possibilities (Tedeschi and Calhoun, 1996). Traumatic events that trigger PTG are often described as “seismic” (Blevins and Tedeschi, in press), disrupting someone's entire way of life and potentially their existing coping strategies. For many people, the COVID-19 pandemic qualifies as a seismic event, given that it not only provokes a health scare, but interrupts the familiar rhythms and patterns of daily life, is impossible to escape, and has made common means of self-distraction like travel or simply going to a pub life-threatening. It has therefore, forced many people to begin to examine alternative ways of life.

Among the participants high in self-transcendent wisdom, we see evidence of such a shift in priorities. Projects oriented around family or immediate communities of friends become more prevalent during physical distancing than they were before. Intrapersonal projects, aimed at some sort of personal change, also become more focal: meditation, yoga, and contemplation on a good life become projects among participants with high self-transcendent wisdom. Memorably, one person showed a newfound commitment to political activism, possibly spurred by the surge in racial justice protests that began during the time of data collection. These projects closely match the pattern of activities that Calhoun et al. (2010) argue best facilitate PTG: reassessing one's goals, strengths, and priorities, sociocultural influences (such as social support and role models), and strategies to manage ongoing stress. They also closely match the conditions that Callard (2018) argues are ideal for supporting intentional self-transformation: ideals, a supportive community, and a sense that such transformation will be meaningful. Previous evidence following the SARS epidemic found that some individuals reported experiencing positive life transformations, similar to PTG (Lau et al., 2006). Blevins and Tedeschi (in press) suggest that PTG reflects an ability for people to respond well to adversity and experience positive changes following the attempt to make sense of a disrupted world. Similar themes to PTG and the conditions for self-transformation have been identified by existential positive psychology in positioning confrontation with periods of suffering as opportunities for cultivating a greater sense of meaning (Wong, 2020). The COVID-19 pandemic has severely disrupted our own worldviews and requires this sense of reconstruction to cope with our new reality.

To use an analogy, the relationship between adaptive coping and self-transcendent wisdom may be compared to boiling water. As one heats a pot of water, at some point, when the liquid water has no more capacity to disperse energy, it turns to steam. People with existing adaptive coping strategies are analogous to a larger pot of water, which takes more heat and longer exposure to boil. Those without such strategies, however, are analogous to smaller pots; the heat represented by the current disruption to daily life is enough to require them not just to cope, but to change.

### Limitations

Like all studies, our study has limitations. The most salient of these is that it captures a single moment in time, representing the experience of North Americans in the early summer of 2020. It is possible that some of our findings might differ were the same questions to be asked of a different sample, or even of the same sample now, in the second winter wave of the pandemic. Another limitation of the present study is that our “baseline” measures from before the pandemic are retrospective, not pre-recorded. While this might open our study up to recency and saliency biases, we feel that this concern is balanced out by the strengths of this retrospective method: a high self-rating of well-being now may be far more meaningful than such a rating a year ago. Importantly, our interest in the present study was predominately idiographic, rather than nomothetic. Our findings should be interpreted as presenting participants' self-understanding of their life and experiences of change during a period of physical distancing and how that has changed, rather than as impersonal objective measures at two points in time. While some researchers were fortunate enough to have baseline measures for their participants that pre-dated the pandemic [see Hamza et al. (2020)], the methods of the present study relied on participant self-understanding in the moment of data collection. Relatedly, it must be remembered that our study is exploratory, not confirmatory. We began this study to investigate a broad assortment of possible influences on how North Americans were spending their time during social distancing as compared to before such measures were enacted, and how this relates to their reported sense of well-being. Our findings, however, repeat a fairly consistent theme in the positive, existential, narrative, and phenomenological psychologies of well-being: feeling connected to others is vital to experiencing a meaningful life.

## Conclusions

The present study used a broad range of measures to explore contributing factors to participants' well-being during physical distancing resulting from the COVID-19 pandemic. In particular, we used retrospective comparison to examine perceived changes in well-being prior to the pandemic, and how they contributed to a change in well-being at the time of the study. We found that self-transcendent wisdom and perceived meaning in life demonstrated the strongest positive associations with change in perceived well-being when controlling for all other variables. Analysis of the personal projects of participants reporting the highest levels of self-transcendent wisdom revealed a pattern consistent with models of PTG and aspirational self-transformation. Our findings suggest that while most participants experienced a decline in well-being, for understandable reasons (e.g., loneliness, negative emotions, and alienation), higher levels of self-transcendent wisdom were associated with positive changes in well-being during physical distancing as compared to before—especially for participants with merely average coping mechanisms, or who belonged to the Substance Use coping profile. Our findings suggest ways to avoid having the COVID-19 pandemic become the traumatic event of a generation, but instead a genuine watershed moment for growth.

## Data Availability Statement

The raw data supporting the conclusions of this article will be made available by the authors, without undue reservation.

## Ethics Statement

The studies involving human participants were reviewed and approved by University of Toronto Research Ethics Board. The participants provided their written informed consent to participate in this study.

## Author Contributions

JK, MM, ZF, and SM designed the study and contributed to writing the manuscript. JK provided theoretical framing and performed qualitative analysis. MM, MA, and RA performed latent profile analysis and data visualization. ZF performed descriptive statistics, multiple regression analysis, and data visualization. SM provided the original concept for the study, performed qualitative analysis, and data visualization. MF provided funding, resources, supervision for the study, and reviewed the manuscript. All authors contributed to the article and approved the submitted version.

## Conflict of Interest

The authors declare that the research was conducted in the absence of any commercial or financial relationships that could be construed as a potential conflict of interest.

## Footnotes

^1^There are 23 responses that came from 11 IP addresses. After examining these responses closely we decided to keep them because it is more likely that responses from the same IP originated from the same household.
